# 4.N. Workshop: Maternal Oral Health Models and Initiatives in Global and Public Health Perspectives

**DOI:** 10.1093/eurpub/ckac129.256

**Published:** 2022-10-25

**Authors:** Organised by: World Federation of Public Health Association Oral Health Workgroup, Chair persons: Hyewon Lee (Switzerland)

## Abstract

Achieving and maintaining good oral health is essential for both the oral and overall health of expecting mothers and the oral health of their young children. Dental caries can negatively affect daily activities, including eating, speaking, and social interaction. When expecting mothers have active dental caries, the risk for dental caries in these children becomes higher. Another common oral disease, periodontitis, was associated with systemic conditions, including cardiovascular disease, respiratory disease, diabetes, and potentially adverse birth outcomes. Some studies also showed the systemic impact of periodontal disease and the related pathogens that can lead to systemic inflammation and adverse birth outcomes, even though periodontal treatment has not been shown to improve birth outcomes. A mother's oral health status, her oral health knowledge, and beliefs also have been shown to significantly affect diet and home oral hygiene practice for young children. All the evidence suggests that pregnancy should be considered a critical period in which oral health education and dental care should be provided to improve the oral health of mothers and their young children. Despite the importance of a mother's oral health and their oral health knowledge in preventing dental caries in young children, maternal oral health is much neglected in primary and prenatal health discussions. This is even more significant in developing countries with inadequate oral health care infrastructure and workforce. In addition, with increased sugar consumption worldwide, oral disease prevention and oral health promotion are the keys to achieving oral health parity among the maternal and child population. This workshop aims to position maternal oral health as essential primary and perinatal health care and discuss the care models and initiatives in various contexts and geographical locations. At the care delivery and community-based interventions, Dr. Sophia will discuss her oral health education and tobacco cessation initiative for pregnant women in India. Dr. Irene will demonstrate her integrated oral health training model for midwives and dental providers in Indonesia. She will describe how her collaboration with the local government achieves collaborative training and care delivery to improve mothers and their spouses’ oral health and oral health knowledge in rural Indonesia. Dr. Jane will showcase her material oral health model for refugee and immigrant mothers in Switzerland. Lastly, Dr. Khabiso from South Africa will present how maternal oral health can be integrated at the national policy level through the collective efforts of public health organizations. Dr. Lee, the chair of this workshop, will provide a literature review on maternal oral health studies and how maternal oral health can be an essential part of primary and perinatal care and the WHO oral health initiative set for 2030.

**Key messages:**

• Maternal oral health is essential in primary and perinatal health care discussion and a critical optic for global and public health agendas.

• There are various maternal oral health programs and initiatives around the globe at care delivery level, community level, and policy level.

